# *PTCH1* gene variants rs357564, rs2236405, rs2297086 and rs41313327, mRNA and tissue expression in basal cell carcinoma patients from Western Mexico

**DOI:** 10.1002/jcla.25010

**Published:** 2024-01-29

**Authors:** Marianela Zambrano‐Román, Jorge R. Padilla‐Gutiérrez, Yeminia Valle, José F. Muñoz‐Valle, Elizabeth Guevara‐Gutiérrez, Diana Emilia Martínez‐Fernández, Emmanuel Valdés‐Alvarado

**Affiliations:** ^1^ Centro Universitario de Ciencias de la Salud, Instituto de Investigación en Ciencias Biomédicas (IICB) Universidad de Guadalajara Guadalajara Mexico; ^2^ Doctorado en Genética Humana, Departamento de Biología Molecular y Genómica Universidad de Guadalajara Guadalajara Mexico; ^3^ Departamento de Dermatología, Instituto Dermatológico de Jalisco “Dr. José Barba Rubio” Secretaría de Salud Jalisco Zapopan Jalisco Mexico

**Keywords:** basal cell carcinoma, genetic variants, mRNA, protein expression, *PTCH1*

## Abstract

**Background:**

Basal cell carcinoma (BCC) represents about 80% of all cases of skin cancer. The PTCH1 is a transmembrane protein of the Sonic Hedgehog signaling pathway that regulates cell proliferation. Genetic variants in *PTCH1* gene have been previously described in association with BCC development. In addition, *PTCH1* mRNA and protein expression analysis are also significant to understand its role in skin cancer physiopathology.

**Methods:**

An analytical cross‐sectional study was performed, and a total of 250 BCC patients and 290 subjects from the control group (CG) were included, all born in western Mexico. The genotypes and relative expression of the mRNA were determined by TaqMan® assay. The protein expression was investigated in 70 BCC paraffin‐embedded samples with PTCH1 antibodies. Semi‐quantitative analysis was performed to determine the expression level in the immunostained cells.

**Results:**

We did not find evidence of an association between *PTCH1* rs357564, rs2297086, rs2236405, and rs41313327 genetic variants and susceptibility to BCC. Likewise, no statistically significant differences were found in the comparison of the mRNA level expression between BCC and CG (*p* > 0.05). The PTCH1 protein showed a low expression in 6 of the analyzed samples and moderate expression in 1 sample. No association was found between genetic variants, protein expression, and demographic‐clinical characteristics (*p* > 0.05).

**Conclusion:**

The studied *PTCH1* variants may not be associated with BCC development in the Western Mexico population. The *PTCH1* mRNA levels were lower in patients with BCC compared to the control group, but its protein was underexpressed in the tissue samples.

## Graphical abstract

There was no association between *PTCH1* genetic variants (rs357564, rs2236405, rs2297086, and rs41313327) and BCC development in the Western Mexico population. *PTCH1* mRNA and protein were underexpressed in BCC patients.

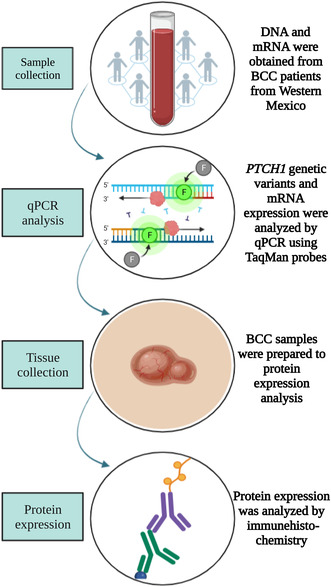

## 1 INTRODUCTION

Non‐melanoma skin cancer (NMSC) represents almost 90% of all cutaneous neoplasms, with more than one million cases per year.^1^, ^2^ Basal Cell Carcinoma (BCC) has the highest prevalence in the NMSC group, which accounts for about 80% of these tumors worldwide, and its incidence keeps rising by nearly 10% each year.^3^ Even though the cancer hallmarks have been broadly defined, the main leading risk factor for developing skin cancer is solar ultraviolet radiation (UVR), which affects the DNA of the cells in the skin epithelium.^4^, ^5^

BCC malignancy spectrum is variable and complex, and it can appear spontaneously or through predisposing genetic syndromes, such as the Nevoid basal cell carcinoma syndrome (NBCCS), due to *PTCH1* gene variants.^6^, ^7^ The *PTCH1* gene is a tumor suppressor located in 9q22.3, containing 24 exons that encodes for a 12‐pass transmembrane glycoprotein called PTCH1, as well.^3^, ^8^ Even though several isoforms have been described early, a longer L isoform (PTCH1b) containing 1447 amino acids with a unique N‐terminal length has been designed as the canonical isoform.^9^, ^10^, ^11^

The Sonic Hedgehog signaling pathway has been recognized as a critical regulator in embryonic development, tissue polarity, cell growth, and finally tumorigenesis when the normal function has been lost.^12^, ^13^ Genetic variants of *PTCH1* have been reported in association with several types of cancer, but relevant with BCC.^14^, ^15^, ^16^, ^17^, ^18^, ^19^ The rs357564 has been analyzed in different populations and several types of cancer, as well as other clinical conditions.^20^, ^21^, ^22^, ^23^ For the rs2297086, rs2236405, and rs41313327 few reports have been published. However, the rs2297086 and rs41313327 have been studied in BCC,^20^, ^24^ and the rs2236405 in reproductive cancers and congenital diseases.^25^, ^26^

Previous studies about *PTCH1* mRNA and protein expression have reported wide differences in expression levels among diverse types of cancer and tissues. This study aimed to analyze the possible association between *PTCH1* gene variants (rs357564, rs2236405, rs2297086, and rs41313327), mRNA, and protein expression in patients with basal cell carcinoma from western Mexico.

## 2 MATERIALS AND METHODS

### 2.1 Subjects

The study group included 250 unrelated patients from Western Mexico with confirmed histopathological diagnosis of basal cell carcinoma (BCC) based on The American Academy of Dermatology (AAD) guidelines^27^ were recruited from the Instituto Dermatológico de Jalisco “Dr. José Barba Rubio” in Guadalajara city, Mexico. On the other hand, 290 unrelated subjects with similar age to the patients were included as a control group (CG). Relative expression of the mRNA was analyzed in BCC patients (*n* = 44) and subjects from the CG (*n* = 44). A separate group of 70 paraffin‐embedded samples were collected for protein expression from BCC patients in the Dermatology Service of Hospital Civil de Guadalajara, following the same selection criteria. All patients were included at the first diagnosis and were not under any treatment for cancer. Both patients and the CG had no history of any known type of cancer or organ transplant. We considered only those subjects with three previous generations, including their own, who had been born in western Mexico, who agreed to participate by signing the informed consent letter.

### 2.2 ETHICS STATEMENT

The study was performed according to the ethical principles for experiments involving humans stated in the World Medical Association Declaration of Helsinki (1964). Ethical approval was obtained by all the involved institution's ethics and research committees: Centro Universitario de Ciencias de la Salud (CUCS) (19–50), Instituto Dermatológico de Jalisco “Dr. José Barba Rubio” (374/2021), Secretaria de Salud Jalisco SSJ/DGEICS/12022, and Hospital Civil de Guadalajara (074/21). Informed consent was obtained from all patients and control group subjects for being included in the study.

### 2.3 Genotyping of the rs41313327, rs357564, rs2297086, rs2236405 variants

Genomic DNA was extracted from peripheral blood leukocytes using Miller's Technique.^28^ The analysis of the variants was by Quantitative Polymerase Chain Reaction (qPCR) using TaqMan probes Assays (catalog 4,351,379): rs41313327 (C__86344157_10), rs357564 (C___3030099_10), rs2297086 (C__16185796_10), and rs2236405 (C__15954310_10), and TaqMan Genotyping Master Mix catalog 4,371,355 (Applied Biosystems™, Foster City, CA, USA) with a Roche LightCycler 96® System. A double‐blind genotyping of 25% of the analyzed samples was performed for all variants as quality control, validating the genotypes.

### 2.4 Relative expression of the mRNA analysis

Total RNA was extracted from peripheral blood leukocytes using TRI Reagent® (SIGMA‐ALDRICH, St. Louis, MO, USA) according to the manufacturer's instruction to obtain total RNA based on the method of Chomczynski and Sacch.^29^ Reverse transcription from 1 μg of total RNA was performed to obtain cDNA following the manufacturer's protocol using Promega (Promega Corporation, USA) reverse transcription reagents M‐MLV Reverse Transcriptase, dNTP Mix, oligo (dT) 15 Primer, RNasin® Ribonuclease Inhibitor and Ribonuclease H. Real‐time qPCR analysis was performed using TaqMan probes *PTCH1* Hs00181117_m1 (catalog 4,448,489) and *GAPDH* Hs02786624_g1 (catalog 4,331,182) according to the conditions indicated in TaqMan Gene Expression Assay Protocol (Applied Biosystems, Foster City, CA, USA) using a Roche LightCycler 96® System. The *GAPDH* gene was used as an internal control to normalize the relative expression, which was evaluated using the 2^−ΔΔCq^ and 2^−ΔCq^ methods, according to Schmittgen and Livak.^30^ Results are presented as a relative fold change compared to the control group and unit relative of expression (URE), respectively. Two replicates were performed for each sample in the relative expression analysis.

### 2.5 PTCH1 protein expression

The paraffin blocks were recovered, and new cuts were made using a Leica Biosystems (USA) brand rotary microtome. Immunohistochemistry was performed using automated staining with the Benchmark ULTRA Roche system. Heat‐induced antigen retrieval was carried out using Cell Conditioning 1 (CC1; Ventana Medical Systems), followed by the application of mouse anti‐PTCH1 monoclonal antibody (catalog GTX83771) at 1:50 dilution, according to the manufacturer's instructions (GeneTx Inc, Irvine, CA, USA). All slides included pancreas‐positive controls, and reactions were detected using the OptiView DAB Detection Kit (Roche) to visualize PTCH1. Hematoxylin–Eosin (HE) staining was used to determine the histopathological subtype.

Intensity and extension due to positive staining cells were the criteria for expression level classification. The staining intensity was classified as 0 (negative staining), 1 (weak staining), 2 (moderate staining), and 3 (strong staining). The positivity was determined by observing all slides at low power (40×) and then randomly selecting three fields at high power (400×). The percentage of positivity was ranked as 0 (no positive cells), 1 (<10% of positive cells), 2 (10%–50% of positive cells), 3 (51%–80% of positive cells), and 4 (>80% of positive cells). The sum of both scores allowed obtaining an expression score from 0 to 7, where 0–3 was considered a low expression and 4–7 was a high expression.

### 2.6 Statistical analysis

The Hardy–Weinberg equilibrium test, genotype, and allele frequencies were calculated by the χ^2^ or Fisher's exact test, when applicable. Prior to data analysis, outliers were removed, as described by David and Hoaglin.^31^ The Mann–Whitney *U*‐test and Kruskal–Wallis test were used to compare differences between groups. The linkage disequilibrium (LD) was calculated using Lewontin's D′ and *r* ^2^. The haplotype analysis was performed by SNPStats (https://www.snpstats.net/start.htm) and SHEsis (http://analysis.bio‐x.cn/myAnalysis.php) software.^32^, ^33^ Odds ratios (OR) and 95% confidence intervals (95% CI) were calculated to test the probability that the genotype and allele frequencies were associated with BCC. A *p*‐value < 0.05 was considered to be statistically significant. All the statistical analyses were done with the SPSS statistical package version 20.

## 3 RESULTS

### 3.1 Clinical and demographic characteristics

The demographic and clinical data of the 250 BCC patients and 290 control group subjects are shown in Table 1. Regarding clinical characteristics, BCC was more frequent in women (62.4%) than men (37.6%). Despite most of the lesions presenting a size greater than 5 mm (70.4%), the majority were classified as low‐risk subtypes (66.8%), where nodular was the principal subtype. However, the infiltrative subtype was also relevant in the high‐risk group. The main location was on exposed anatomic areas such as the head and neck (86.4%). A significant difference was found between histological subtype and location (*p* = 0.04).

**TABLE 1:** Demographic and clinical characteristics.

Demographic characteristics			
Variable	BCC (*n* = 250) *n* (%)	CG (*n* = 290) *n* (%)	*p*
Age	67 (26–96)^a^	65 (25–92)^a^	0.115
Sex			
Female	156 (62.4)	158 (54.5)	0.899
Male	94 (37.6)	132 (45.5)	
Clinical and histological characteristics			
Histological subtype			
Low‐risk	167 (66.8)		
High‐risk	66 (26.4)		
Mixed^b^	17 (6.8)		
Size			
>5 mm	176 (70.4)		
<5 mm	74 (29.6)		
Localization			
Head and neck	216 (86.4)		
Trunk	21 (8.4)		
Arms	4 (1.6)		
Legs	2 (0.8)		
Genital	1 (0.4)		
More than 1 lesion	6 (2.4)		

Abbreviations: BCC, basal cell carcinoma; CG, control group.

^a^ The data were expressed as mean (range).

^b^ Corresponds to low‐ and high‐risk subtypes simultaneously.

### 3.2 Genotype and allele analysis

The distribution of the genotypes in the control group was found in Hardy–Weinberg equilibrium (HWE) for the variants rs357564 (*p* = 0.63) and rs2236405 (*p* = 0.86). The variants rs2297086 (G > A) and rs41313327 (C > T) were found as monomorphic variants with the presence of the major allele in all the genotyped samples (alleles G and C, respectively). Due to this, the HWE was not achieved (*p* < 0.05).

For the rs357564 variant, the A allele was shown with similar frequency among the CG and BCC patients, and the G/A genotype was the most frequent in both groups (51.4% and 52.8%, respectively). On the other hand, for the rs2236405 variant, the A/A genotype was not found in either of the studied groups. The minor allele (A) was only present in the heterozygous state with a frequency lower than 2%. No statistically significant differences were found in evaluating the genetic models or the allelic and genotypic frequencies (Table 2).

**TABLE 2:** Allele and genotype distribution of the *PTCH1* gene variants.

	CG *n* (%)	BCC *n* (%)	OR CI (95%)	*p*
rs357564				
Genotype				
G/G	73 (25.2)	65 (26)	1	–
G/A	149 (51.4)	132 (52.8)	0.995 (0.661–1.496)	0.980
A/A	68 (23.4)	53 (21.2)	0.875 (0.536–1.430)	0.594
Allele				
G	295 (50.8)	262 (52.4)		
A	285 (49.2)	238 (47.6)	0.94 (0.740–1.195)	0.614
Dominant model				
GG	73 (25.2)	65 (26)	1	–
GA + AA	217 (74.8)	185 (74)	1.04 (0.71–1.54)	0.83
Recessive model				
GG + GA	222 (76.6)	197 (78.8)	1	–
AA	68 (23.4)	53 (21.2)	1.14 (0.76–1.71)	0.53
rs2236405				
Genotype				
T/T	284 (97.9)	241 (96.4)	1	–
T/A	6 (2.1)	9 (3.6)	1.768 (0.620–5.037)	0.280
A/A	0 (0)	0 (0)	–	–
Allele				
T	574 (99)	491 (98.2)		
A	6 (1)	9 (1.82)	1.754 (0.620–4.961)	0.283
rs2297086				
Genotype				
G/G	290 (100)	250 (100)	1	–
G/A	0 (0)	0 (0)	–	–
A/A	0 (0)	0 (0)	–	–
Allele				
G	580 (100)	500 (100)		
A	0 (0)	0 (0)	–	–
rs41313327				
Genotype				
C/C	290 (100)	250 (100)	1	–
C/T	0 (0)	0 (0)	–	–
T/T	0 (0)	0 (0)	–	–
Allele				
C	580 (100)	500 (100)		
T	0 (0)	0 (0)	–	–

Abbreviations: BCC, basal cell carcinoma; CG, control group; CI, confidence interval; OR, odds ratio.

### 3.3 Haplotype analysis of *PTCH1*

For haplotype analysis, we excluded the rs2297086 (G > A) and rs41313327 (C > T). The variants rs357564 (G > A) and rs2236405 (T > A) were found in high linkage disequilibrium (D′ = 0.99, *r* ^2^ = 0.013, *p* < 0.001). The major haplotype was GT, accounting for 50.6% and 49.8% in BCC patients and CG, respectively. However, no significant differences in haplotype distribution were detected (Table 3).

**TABLE 3:** *PTCH1* haplotype distribution in BCC patients and control group.

Haplotype	BCC *n* (%)	CG *n* (%)	OR (CI 95%)	*p*
GT	253 (50.6)	289 (49.8)	1	–
AT	238 (47.6)	285 (49.1)	1.05 (0.82–1.35)	0.69
GA	9 (1.8)	6 (1.1)	0.58 (0.20–1.66)	0.31

*Note*: Haplotype is represented by rs357564 and rs2236405 variants.

Abbreviations: BCC, basal cell carcinoma; CG, control group; CI, confidence interval; OR, odds ratio.

### 3.4 *PTCH1* mRNA expression

**FIGURE 1:**
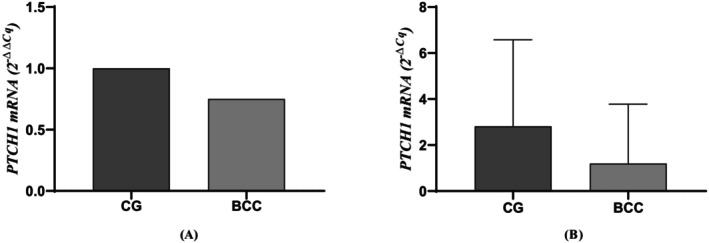
Comparison of *PTCH1* mRNA expression. (A) Comparison of BCC patients and CG by the 2^−ΔΔCq^ method. (B) Comparison of BCC patients and CG by the 2^−ΔCq^ method. BCC, basal cell carcinoma; CG, control group.

The mRNA expression of *PTCH1* was only detected in 22 of the analyzed BCC samples and 14 of the CG subjects. BCC patients exhibited lower median mRNA expression in peripheral blood leukocytes compared to CG (1.3‐fold less). However, the median of *PTCH1* mRNA was 1.20 URE in BCC patients, while in the CG was 2.81 URE, with no significant results (*p* > 0.05) (Figure 1). Next, BCC patients were stratified according to rs357564 genotypes and clinical characteristics. We did not observe differences in mRNA expression according to sex, location, lesion size, and histopathological risk grade, as well as genotypes and dominant and recessive models of inheritance.

### 3.5 PTCH1 expression in BCC tissue

Following a systematic protocol, the expression was double‐blind analyzed by two independent observers. For this group, women were also representative of 62.9%. The most common histological subtype was nodular (50%), followed by micronodular (14.3%), morpheiform (14.3%), adenoid cystic (8.6%), metatypic (5.7%), nodulocystic (5.7%), and superficial (1.4%). Inflammation occurred in almost all patients (95.7%), and the most frequent Clark level was IV (45.7%), but this feature was not possible to determine in four samples. Most patients had solar elastosis (85.7%), desmoplasia was mainly moderate (52.9%), and the pigment was present in 52.9% of the samples.

**FIGURE 2:**
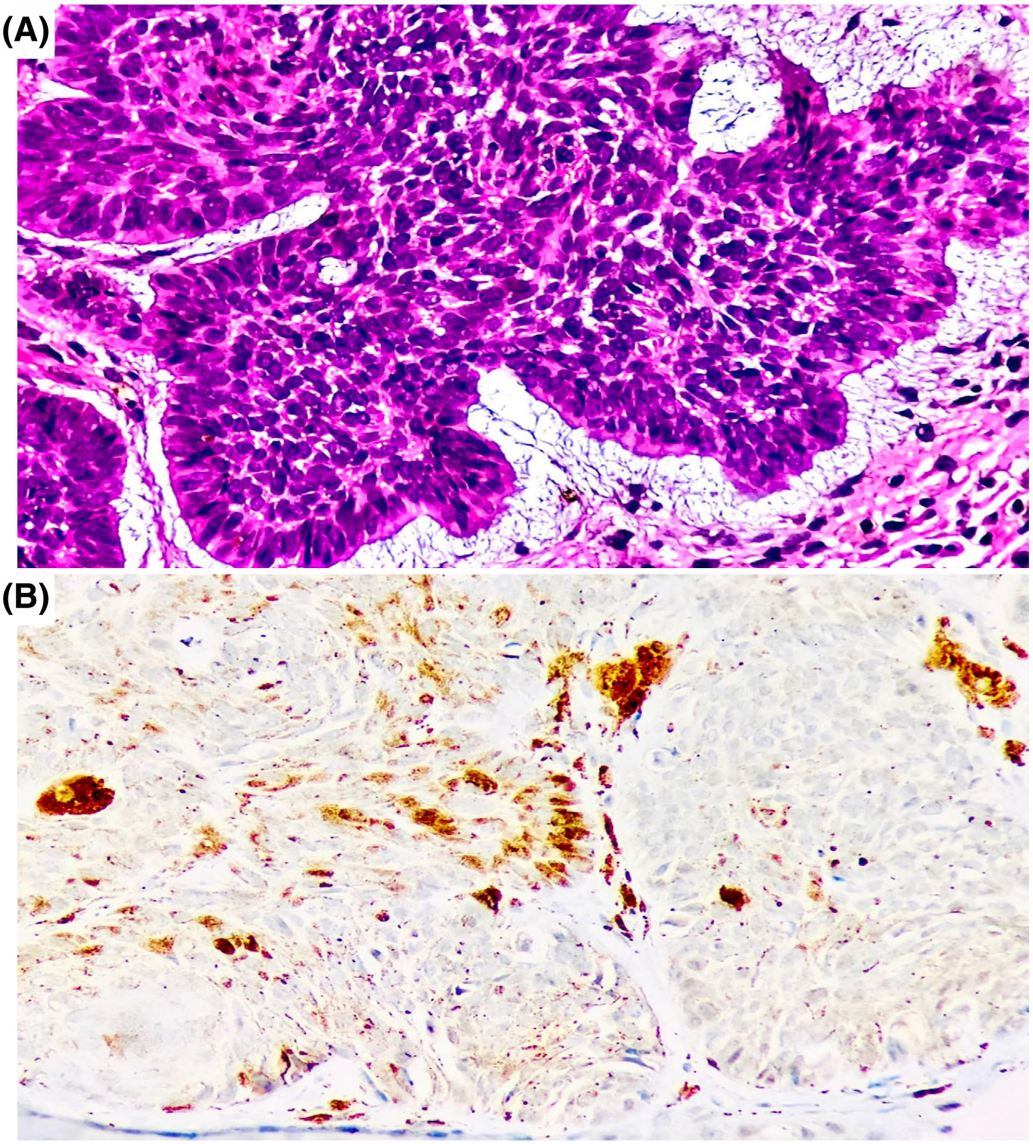
PTCH1 protein expression. (A) H&E staining image of nodular BCC, the most frequent subtype within the study group. Neoplastic cells are characterized by basaloid appearance, always arranged in a palisade. (B) Positive immunohistochemistry staining for PTCH1 protein, with a low expression prevailing in the low‐risk subtypes, and negative expression in high‐risk subtypes.

PTCH1 expression was detected in 7/70 analyzed samples, six had a low‐expression and one had moderate expression. The PTCH1 positive histological subtypes were essentially nodular, cystic‐adenoid, micronodular and cystic‐nodule, while more aggressive subtypes such as morpheiform and metatypical were negative (Figure 2). There was a significant difference related to inflammation between the patients with or without PTCH1 expression (*p* < 0.001).

### 3.6 Logistic regression analysis

In the logistic regression analysis, the characteristics were designated sex, age (<60 years vs >60 years), histopathology, location and genotypes of the rs357564 variant as independent variables and the size of the lesions as the dependent variable. It was observed that patients aged ≥60 years have a higher risk of presenting lesions with sizes greater than 5 mm (OR = 3.289, 95% CI 1.70–6.35, *p* = <0.001) (Table 4).

**TABLE 4:** Logistic regression analysis.

Equation variables								
Independent variables	β^a^	S.E.^b^	Wald^c^	D.F.^d^	*p*‐Value	OR^e^	95% CI^f^	
							Low	High
Sex								
Female (ref)								
Male	0.427	0.312	1.872	1	0.171	1.533	0.83	2.82
Age								
<60 years (ref)								
>60 years	1.191	0.336	12.56	1	**0.0001**	3.289	1.70	6.35
Histopathology								
Low (ref)			3.128	2	0.209			
High	0.437	0.347	1.588	1	0.208	1.548	0.78	3.05
Mixed subtypes	1.100	0.804	1.871	1	0.171	3.003	0.62	14.51
Location								
Trunk (ref)								
Head and neck	−0.559	0.768	0.529	1	0.467	0.572	0.12	2.57
Codominant model								
GG (ref)			3.845	2	0.146			
GA	0.580	0.373	2.415	1	0.120	1.785	0.86	3.70
AA	−0.060	0.418	0.020	1	0.887	0.942	0.41	2.13
Constant	−0.083	0.801	0.011	1	0.918	0.920		

*Note*: Adjusted by size (0 = <5 mm, 1 = >5 mm). Results with significant association (*p* < 0.05) are emphasized by the bold values.

^a^ Regression coefficient.

^b^ Standard error.

^c^ Wald test.

^d^ Degrees of freedom.

^e^ Odds ratio.

^f^ Confidence interval.

## 4 DISCUSSION

Skin cancer's incidence continues to increase, and UVB radiation is known to cause DNA lesions that affect replication and transcription processes, if they are not properly repaired.^34^, ^35^ The BCC is the most frequent within this group representing 80% of all the cases.^36^ Our findings indicated a higher prevalence of BCC in women. However, previous reports consider that men are more frequently affected, especially when they are older than 50 years old,^37^ in congruence with our group's reported age. The prevalence observed among females may be associated with an aesthetic issue that promotes the search for medical attention.

Although clinical features allow detecting a malignant lesion, the histopathological study is essential for diagnosis confirmation, and even treatment decisions.^38^ Low‐risk subtypes such as superficial, pigmented, nodular, and adenoid are the most frequent. Nevertheless, high‐risk lesions are more aggressive, and mixed subtypes may be difficult to treat.^39^, ^40^ Since a significant difference between the histological subtype and the location was found, we propose that unprotected skin areas are predisposed to developing high‐risk lesions due to continuing UV sunlight exposure. Besides, as the size of the lesions is larger, the prognosis may be worse, and the possibility of further recurrence remains to increase.

The Sonic Hedgehog signaling pathway has an essential role in cell proliferation control. Its association with non‐melanoma skin cancer arises from genetic syndromes in which patients are known to develop BCC since childhood. However, *PTCH1* gene function may be altered in 70% to 80% of sporadic BCC cases, and the presence of a genetic variants might be a determinant.^18^, ^19^

The rs357564 variant has been broadly analyzed in several clinical conditions.^14^, ^15^, ^22^, ^41^ Although no statistical significance was found regarding allele and genotype frequency distribution, the G/A genotype was representative in both study groups (BCC and CG). Previously, studies in patients with multiple lesions reported a higher frequency of the G/G genotype.^42^, ^43^ Nonetheless, Strange et al.^44^ reported the prevalence of the G/A genotype but with no statistical significance, in congruence with our findings. The presence of minor allele (A) with differences between cases and control group has also been reported, suggesting that the proline change to leucine amino acids in codon 1315 may have a relevant role in cancer development.^16^

The minor allele (A) of the rs2236405 variant has been reported at a low frequency (∼2%). In our study groups, it was identified with a similar frequency and only in the heterozygous state (T/A). Regarding the rs2297086 variant, despite the frequencies reported in several populations for the minor allele (A), the frequency distribution in our study population indicated 100% for the G allele. Previous studies analyzed the rs2297086 and rs357564 in BCC patients with a history of transplants, in which an increased risk of developing this type of lesions has been described. Even though the frequency of the A allele (rs357564) was represented in the group of cases compared to the controls, no statistically significant differences were found in the Italian population.^20^

The background of rs2297086 is limited, and the impact of intronic variants may sometimes seem uncertain. Possibly, they are involved in a regulatory manner, especially in those cases where their location is not adjacent to the exon‐intron boundary, or they could even generate alternative splicing sequences.^45^ Finally, the genotyping of the rs41313327 (C > T) demonstrated the prevalence of the homozygous genotype for the wild allele (C/C), following the frequencies reported in databases and even with previous investigations, in which patients with clinical conditions—other than cancer—had a similar frequency of the C allele to our studied groups, where the presence of the minor allele (T) was not detected either.^46^ However, previous studies in BCC patients from the Polish population reported the frequencies of the C/C, C/T, and T/T genotypes with no differences between patients and controls.^24^

We also investigate a possible pathogenic effect in PROVEAN, SIFT, Polyphen, and FATHMM due to aminoacid change for exonic variants rs357564, rs2236405, and rs41313327 or splicing effect of intronic variant rs2297086 in regSNP‐intron and Human Splicing Finder.^47^, ^48^, ^49^, ^50^, ^51^, ^52^ Although it was not conclusive, we could presume that the rs357564 may have a deleterious effect as we consider that the c‐terminal domain is related to SMO protein retention. On the other hand, intronic variant rs2297086 did not show an association with splicing signs or the creation of a new branch point in the intron (data not shown).

To our knowledge, this is the first report about linkage disequilibrium (LD) between the variants rs357564‐rs2236405 in BCC patients. There are few reports where the rs2236405 is in LD with other *PTCH1* variants, providing a decreased risk in patients with reproductive cancers.^25^ While haplotype analysis of the rs357564 variant was associated with an increased BCC risk in patients with previous transplants.^20^ Even though our results did not show statistically significant differences, we recognize the importance of studying these variants and knowing that other variants of the *PTCH1* gene could be involved in BCC pathogenesis in our population.

Regarding the analysis of the relative expression of *PTCH1* mRNA, no significant differences were found between BCC and CG (*p* > 0.05). No expression was detected either in peripheral leukocytes or tissue from BCC patients with high‐risk subtypes. Given its role as a tumor suppressor, it is possible to consider that an altered regulation promotes cell proliferation and participates in the progression of aggressive lesion types.^53^ Although there is a lack of *PTCH1* expression studies from peripheral blood leukocytes, mRNA expression from tumor tissue describes an overexpression in various types of cancer and even a significant correlation with metastasis, resistance to therapy, and advanced stages of the disease. A recent study indicated a similar immunoreactivity of PTCH1 in BCC and Merkel carcinoma, where the expression was highly significant to healthy skin expression.^54^

In this sense, mRNA analysis in different types of cancer has shown diverse results. García‐Martínez et al.^55^ reported the *PTCH1* mRNA overexpression in cell lines derived from mammary basal cells. In contrast, mRNA analysis of the elements of the Hedgehog signaling pathway, such as *SHH*, *SMO*, and *GLI1* in tissue from renal cell carcinoma showed higher expression levels than healthy tissue, while an underexpression of *PTCH1* was observed and specifically in patients older than 60 years.^56^ Besides, Kim et al.^57^ described an elevated expression of *PTCH1* mRNA in BCC. However, when compared with tissue protein expression, there were inconsistencies because of a lower expression, and no significant differences between different types of skin tumors. The authors suggested that this may not be surprising since mRNA and protein expression are biologically affected by post‐transcriptional modification and different degradation rates.

Regarding the above‐mentioned, our results about protein expression in BCC tissue, despite being in independent groups, maintain consistency with mRNA expression results, in patients with similar age and histological subtypes. Previous reports suggested that low‐risk subtypes expressed PTCH1 protein more frequently,^3^ in concordance with our findings. In both of the analyzed groups of patients with BCC, mRNA and protein expression was less frequent in high‐risk subtypes such as infiltrative lesions. This may be indicative of a lack of activity that is leading to dysfunctional Hedgehog signaling. We cannot deny that other mechanisms, such as methylation or the activation of non‐canonical Hedgehog signaling could be involved. The mRNA analysis from peripheral blood leukocytes has been poorly reported,^58^ and we recognize the importance of analyzing the proper matrix in the expression level determination.

In conclusion, the proposed genetic variants (rs357564, rs2236405, rs2297086, and rs41313327) of the *PTCH1* gene may not be involved in basal cell carcinoma development in the western Mexico population. However, the possibility that other *PTCH1* variants have an essential role in the pathogenesis cannot be excluded. The observed differences among populations are due to genetic variability. We recognize that sample size could be a limitation in obtaining proper allelic and genotypic distribution, especially in variants with rare frequency, as well as in the analysis of the mRNA and protein expression in the tissue. The examination of clinical and histological characteristics is essential for adequate patient management. The *PTCH1* mRNA levels were lower in patients with BCC compared to the control group, but an underexpression of its protein was found in the tumor tissue, which may be related to a Hedgehog deficient signaling that promotes cell growth and proliferation.

## AUTHOR CONTRIBUTIONS

Marianela Zambrano‐Román: Conceptualization, Formal analysis, Investigation, Visualization, Writing‐original draft preparation. Jorge R. Padilla‐Gutiérrez: Conceptualization, Writing – review and Editing, Yeminia Valle: Conceptualization, Writing – review and Editing, José F. Muñoz‐Valle: Visualization, Supervision, Elizabeth Guevara‐Gutiérrez: Investigation, Visualization, Supervision, Diana Emilia Martínez‐Fernández: Formal analysis, Writing‐review and editing, and Emmanuel Valdés‐Alvarado: Conceptualization, Formal analysis, Investigation, Visualization, Writing‐original draft preparation, Funding acquisition. All authors have read and agreed to the published version of the manuscript.

## FUNDING INFORMATION

This work was supported by PIN 2020 CUCS‐UDG.

## CONFLICT OF INTEREST STATEMENT

The authors declare no conflict of interest.

## Data Availability

All data and reagents are available upon request.
